# Antibiotic-Resistant Bacteria Are Major Threats of Otitis Media in Wollo Area, Northeastern Ethiopia: A Ten-Year Retrospective Analysis

**DOI:** 10.1155/2016/8724671

**Published:** 2016-01-19

**Authors:** Ayele Argaw-Denboba, Asrat Agalu Abejew, Alemayehu Gashaw Mekonnen

**Affiliations:** ^1^Department of Experimental Medicine and Surgery, University of Rome Tor Vergata, Via Montpellier 1, 00133 Rome, Italy; ^2^Department of Pharmacy, College of Medicine and Health Sciences, Wollo University, P.O. Box 1145, Dessie, Ethiopia; ^3^Microbiology Unit, Dessie Regional Health Research Laboratory, P.O. Box 686, Dessie, Ethiopia

## Abstract

Antibiotic resistance is an increasingly serious threat to human health that needs an urgent action. The aim of this study was to determine the prevalence and antibiotic susceptibility profiles of bacteria isolated from patient ear discharges suspected of otitis media. A retrospective analysis was performed using culture and antibiotic susceptibility test results of 1225 patients who visited Dessie Regional Health Research Laboratory from 2001 to 2011. Results showed a strong association (*P* < 0.001) between age and the risk of acquiring middle ear infection. The predominant bacterial isolates were* Proteus* spp. (28.8%),* Staphylococcus aureus* (23.7%), and* Pseudomonas* spp. (17.2%). Most of the isolated bacteria showed high resistance to ampicillin (88.5%), ceftriaxone (84.5%), amoxicillin (81.9%), and tetracycline (74.5%). About 72.5% of* Proteus* spp. and 62.2% of* Pseudomonas* spp. have developed resistance to one and more antibiotics used to treat them. This retrospective study also revealed the overall antibiotic resistance rate of bacterial isolates was increased nearly twofold (*P* = 0.001) over the last decade. Relatively, ciprofloxacin and gentamicin were the most effective antibiotics against all the isolates. In conclusion, antibiotic-resistant bacteria are alarmingly increasing in Wollo area, northeastern Ethiopia, and becoming a major public health problem in the management of patients with middle ear infection.

## 1. Introduction

Ear infection is a common clinical problem throughout the world and the major cause of preventable hearing loss in the developing world [1, 2]. Microbial agents can infect the middle and external parts of the ear and may involve the skin, cartilage, periosteum, ear canal, and tympanic and mastoid cavities [3]. Ear infection can be classified as acute suppurative otitis media (ASOM), chronic suppurative otitis media (CSOM), or otitis externa (OE) [4]. Its chronic form is a serious problem in all age groups with less chance of recovery. In certain cases this condition can lead to serious life-threatening complications, such as hearing impairment, brain abscesses, or meningitis, mostly in childhood and late in life [2, 4]. According to the World Health Organization (WHO) estimates of 2015 over 5% of the world's populations (328 million adults and 32 million children) have disabling hearing loss. The highest prevalence is found in the Asia-Pacific, South Asia, and Sub-Saharan African regions. Half of all cases of hearing loss are avoidable through primary prevention while many can be treated. A leading cause of hearing loss in younger ages, particularly in low- and middle-income countries, is untreated ear infections, often with discharge from the ear. Vaccine-preventable infectious diseases such as rubella, meningitis, measles, or mumps can also lead to hearing loss [2, 4].

The causative agents of ear infection might be bacterial, viral, or fungal. However, the major causative agents of ear infection are bacterial isolates such as* Pseudomonas aeruginosa*,* Escherichia coli*,* S. aureus*,* Streptococcus pyogenes*,* Proteus mirabilis*,* Klebsiella* spp., or mixed bacterial infection [5]. The microbiological profiles of ear infection are well documented in developed world. However, so far few studies have been conducted in most developing countries [6–10]. Moreover, the signs and symptoms of earache may often mislead the etiology of the infection, which makes it very difficult for the clinician to relate the disease to the exact etiology. Hence, the physician may advocate antibiotic therapy irrespective of the etiology of the disease. This may lead to unwanted economic loss, stress to the patient if the ear infection is due to the virus or fungi, and foremost antibiotic resistance [11]. For these reasons, it is very important to study the microbiological profiles of ear infection and their extent of antibiotic resistance in those developing countries for the proper management of patients with ear infections. Moreover, antibiotic resistance is a growing global problem listed among major threats to human health by the World Health Organization [10].

Ethiopia is among the least developing countries and there are only a handful of studies reported so far that showed the prevalence and antibiotic resistance profile of bacterial isolates associated with ear infections [12–19]. Therefore, in this study, we showed a ten-year retrospective analysis of culture and antibiotic susceptibility test results of middle ear discharge from patients suspected of having a middle ear infection and referred to Dessie Regional Health Research Laboratory (DRHRL), located in Dessie town, northeastern Ethiopia.

## 2. Methods

### 2.1. Study Design, Site, and Data Collection

This retrospective study was conducted from June 1 to August 31, 2012. During this period we reviewed culture and antibiotic susceptibility test results of middle ear discharges from 1225 patients suspected of middle ear bacterial infection and referred to DRHRL from September 2001 to September 2011 for antimicrobial sensitive test. DRHRL is located in Dessie town, South Wollo Zone, northeastern Ethiopia. This health research laboratory is the only regional referral laboratory found in the northeastern parts in Ethiopia serving populations of more than 4 million surrounding it [20]. Patient data were collected by the principal investigators using retrospective chart review data collection method based on the articulated aims of this study. Thus, all patient information registered in microbiology laboratory unit patient registration books from September 2001 to September 2011 were collected, including patient sociodemographic characteristics, isolated bacteria from middle ear discharge, and their antibiotic susceptibility test results categorized in each year of the study period. Of note, data with incomplete records or illegible handwriting were excluded from this study.

### 2.2. Laboratory Diagnostic Methods

As the standard operational procedure of DRHRL microbiology unit shows, ear discharge specimens were taken aseptically using sterilized cotton swabs from each patient; then specimens were inoculated on MacConkey agar, blood agar, mannitol salt agar, and chocolate agar plates using sterilized 0.001 mL inoculation loop (all media from Oxoid Limited, UK). The plates of MacConkey, blood, and mannitol salt agar were placed in an aerobic incubator while the chocolate plate was incubated in a carbon-dioxide enriched atmosphere at 37°C for 24 hours. The swarming feature of* Proteus* spp. was managed by subculturing mixed colonies into MacConkey agar that has bile salt and by adding 5% of 90% ethanol. Bacterial isolation and identification were performed according to standard microbiological methods as described in the notable book of Monica Cheesbrough [21]. In brief, Gram-negative bacteria were identified by performing a series of biochemical tests, namely, triple sugar iron agar, indole, Simon's citrate agar, lysine iron agar, urea, mannitol, and motility. Gram-positive bacteria were identified based on their Gram reaction, novobiocin, catalase, and coagulase test results.

Antimicrobial susceptibility tests were performed on Mueller-Hinton agar (Oxoid Limited, UK) using modified Kirby-Bauer disc diffusion method [22]. The susceptibility pattern of each bacterial isolate was interpreted according to the standard criteria of Clinical and Laboratory Standards Institute (CLSI, 2011). The antimicrobial agents tested were amoxicillin (10 *μ*g), tetracycline (30 *μ*g), TMP-SMX (25 *μ*g), cephalothin (30 *μ*g), ampicillin (10 *μ*g), chloramphenicol (30 *μ*g), ceftriaxone (30 *μ*g), ciprofloxacin (5 *μ*g), doxycycline (30 *μ*g), erythromycin (15 *μ*g), gentamicin (10 *μ*g), cefotaxime (30 *μ*g), kanamycin (30 *μ*g), nitrofurantoin (300 *μ*g), vancomycin (30 *μ*g), carbenicillin (100 *μ*g), and clindamycin (2 *μ*g) (Oxoid Limited, UK). To assure the accuracy and reliability of antimicrobial susceptibility test, the reference strains* S. aureus* (ATCC25923),* Escherichia coli* (ATCC25922), and* P. aeruginosa* (ATCC 27853) were used as internal quality controls.

### 2.3. Statistical Analysis

Data were checked for completeness, cleaned manually, entered, and analyzed using SPSS version 20 statistical software and Excel. The chi-square test (*χ* ^2^) was used to measure the association between sex and age with susceptibility to middle ear infection and paired Student's *t*-test was applied to compare antimicrobial resistance rates between the start and end of the study period. The *P* value of < 0.05 was considered to show a statistically significant difference.

### 2.4. Ethical Considerations

Ethical clearance was obtained from Ethical Review Committee of Wollo University. A formal supportive letter was also obtained from the Wollo University to the head of Dessie Regional Health Research Laboratory for cooperation and permission to get the ten-year data record. Patient privacy was protected by deidentification of records. Names of patients were replaced by initials. All data obtained in the course of the study were kept confidential and used only for this study.

## 3. Results

### 3.1. Age and Sex Distribution of Patients Suspected of Otitis Media

After the analysis of 1225 ear discharge culture results of suspected patients, about 1024 (83.6%) were found positive for one or more bacterial species. The result displayed in Table 1 shows both men and women were at equal risk of acquiring middle ear infection by 50.4% and 49.1%, respectively. The frequency of positive ear discharge cultures was higher in the age group of 16–35 years (42.4%) followed by the age group 5–15 years (24.7%) (Table 1). The chi-square test showed age and frequency of bacterial positive ear discharge in each year of the study period were strongly associated (*P* < 0.001).

### 3.2. Prevalence of Isolated Bacteria from Ear Discharges

Out of 1024 bacteria positive patient ear discharge specimens' 9 different pathogenic bacteria species were identified. Among these bacterial species, the predominant bacterial isolate was* Proteus *spp. 324 (28.8%) followed by* S. aureus *266 (23.7%) and* Pseudomonas *spp. 193 (17.2%) (Table 2).

### 3.3. Overall Antibiotic Susceptibility Profiles of Isolated Bacteria from Ear Discharge

During the ten-year period 17 different antimicrobial agents were used to test the antibiotic susceptibility patterns of the pathogenic bacteria isolated from ear discharges. The overall susceptibility profiles of bacterial isolates are shown in Table 3. Out of the total antibiotics examined during the ten-year period, ampicillin had the highest overall resistance rate (88.5%) followed by ceftriaxone (84.5%), amoxicillin (81.9%), and tetracycline (74.5%). Conversely, majority of bacterial isolates were susceptible to ciprofloxacin, gentamicin, and nitrofurantoin with overall resistance rates of 6%, 17.2%, and 21.5%, respectively.

### 3.4. Species Specific Antibiotic Susceptibility Profile of Pathogenic Bacteria Isolated from Ear Discharges

Species specific antibiotic susceptibility profiles are displayed in Table 4.* Proteus* spp., the most frequently isolated bacterium, showed high resistance rate (>74%) to each of the following antibiotics: amoxicillin, tetracycline, ampicillin, chloramphenicol, ceftriaxone, doxycycline, erythromycin, cefotaxime, and vancomycin. Likewise,* S. aureus* showed a resistance rate of (>52%) to each of the following antibiotics: amoxicillin, tetracycline, ampicillin, and cefotaxime.* Pseudomonas* spp., the third most common isolates, exhibited resistance rates of (>73%) to each of the following antibiotics: amoxicillin, tetracycline, TMP-SMX, ceftriaxone, doxycycline, erythromycin, and cefotaxime.* E. coli* had also showed resistance rates of more than 63% for each of the following antibiotics: amoxicillin, tetracycline, ampicillin, doxycycline, and erythromycin. In contrast, almost all the isolated pathogenic bacteria were susceptible to ciprofloxacin and gentamicin with resistance rates of 0%–10% and 2%–25%, respectively.

### 3.5. Multi-Antibiotic-Resistant Pathogenic Bacteria Isolated from Ear Discharges

**Figure 1:**
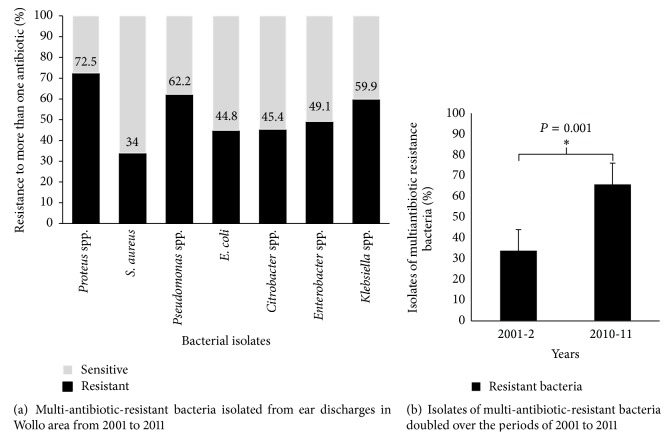
Rates of multi-antibiotic-resistant pathogenic bacteria isolated from patients suspected of having otitis media.

Almost all the isolated bacteria were found to be resistant to one and more of the commonly used antibiotics (Figure 1(a)). Among the total* Proteus* spp. isolated, the majority (72.5%) of the isolates have developed resistance to one and more antibiotics in clinical use. Similarly, about two-thirds of (62.2%)* Pseudomonas* spp. isolates were able to resist one and more antibiotics commonly used to treat them. About 34%–59.9% of the other bacterial isolates have also showed overall antibiotic resistance to one and more antibiotics used in clinics. Comparing the overall antibiotic resistance rate of bacterial isolates from start to end of the study periods (2001-2 versus 2010-11) the resistance rate to one and more of the antibiotics was increased nearly twofold (34% versus 66%, *P* = 0.001) (Figure 1(b)).

## 4. Discussion

Ear infection is a more frequent treatable health care problem worldwide, yet if left untreated, it can cause a serious complication such as a speech development disorder, hearing loss, distress in patients and their family quality of life, and economic burden on the health care system [2]. The burden and prevalence of ear infection are more intense in developing countries due to the poor living standard and hygienic conditions along with lack of proper nutrition [5, 19, 23]. Thus, highlighting the etiologies of ear infection and their antibiotic susceptibility pattern will help to lessen the severe complication of the infection and guide the empirical antibiotic prescribed by the physicians, especially for developing countries [19, 24]. On top of these issues, increased antimicrobial resistance is one of the greatest global public health challenges, which has been accelerated by overprescription of antibiotics worldwide. Infection with antibiotic-resistant bacteria may cause severe illness, increased mortality rates, and an increased risk of complications and admission to hospital and longer stay [24–26]. In light of these facts, this study revealed that* Proteus* spp.,* S. aureus*,* Pseudomonas* spp., and* E. coli* were the most prevalent multi-antibiotic-resistant pathogenic bacteria isolated from suspected patient ear discharges with otitis media in Wollo area, the northeastern part of Ethiopia.

This ten-year retrospective analysis revealed that gender has no influence on the risk of acquiring middle ear infection. Likewise, most investigators have reported no clear gender based difference exists in the risk of acquiring middle ear infection [23, 27]. However, we found that age has a strong association with the risk of acquiring middle ear infection. Similar to our finding other previously reported data from Ethiopia and many other countries also showed age has significant influence on the risk of acquiring middle ear infections [18, 27]. For example, children are highly vulnerable to frequent ear infection due to pathogenic bacteria colonization in the middle ear or upper respiratory tract [12, 19, 28–30].

In this study, the main pathogenic bacterium associated with middle ear infection was* Proteus* spp. followed by* S. aureus* and* Pseudomonas* spp., respectively. Similarly, previously published articles from our study area and other parts of Ethiopia also reported* Proteus* spp. were the foremost bacteria associated with middle ear infection followed by the later ones [12–19]. Although it needs a further nationwide study, taking into account our study finding and others, it seems that* Proteus* spp. is the leading bacterial isolates associated with middle ear infection in Ethiopia. Conversely, several other published data pieces from Africa and elsewhere in the world reported* Pseudomonas* spp., mainly* P. aeruginosa*, is the primary pathogenic bacteria associated with middle ear infection [5, 31–37]. One possible explanation for this difference might be due to climate and geographical variations between Ethiopia and those countries. It is noteworthy that this study and many other previously reported data indicated* S. aureus* is the second common bacterial isolates that often associated with middle ear infection [15, 18, 32, 34, 37].

Ear infection is among the most common illnesses that leads to overprescription of antibiotic use, one of the reasons for the emergence of antibiotic resistant pathogenic bacteria [23, 24]. In light of this evidence, our study revealed that most of the isolated pathogenic bacteria have become resistant to all the easily available antibiotics. In general, ampicillin, ceftriaxone, amoxicillin, and tetracycline had shown the highest antibiotic resistance rates to all bacterial pathogens isolated from middle ear discharge, respectively. Correspondingly, tetracycline, ampicillin, ceftriaxone, and erythromycin were the most clinically used antibiotics that showed a higher resistance rate to* Proteus* spp., which is in line with earlier reports from Ethiopia, Nigeria, and Egypt [17, 18, 38, 39]. Likewise, amoxicillin and ampicillin showed higher resistance rates to* S. aureus*; similar high percentage of resistance was also reported from Ethiopia and elsewhere in the world [5, 17, 18, 40, 41]. Amoxicillin, TMP-SMX, and erythromycin also showed a high level of resistance to* Pseudomonas* spp.; these findings are consistent with other studies [17–19, 42]. Among the total* Proteus* spp. and* Pseudomonas* spp. isolated during the ten-year period about 72.5% and 62.2% of the isolated bacteria have developed resistance to one and more antibiotics that were once in clinical use, respectively. Overall, more than half of the bacterial isolates of this study were characterized as multi-antibiotic-resistant pathogenic bacteria. The reason for this high degree of multiantibiotic resistance might be linked to indiscriminate use of antibiotics, including animal husbandry, self-medication, and poor infection prevention and control practices as indicated by the recent WHO antimicrobial resistance report and earlier studies [10, 19, 43, 44].

Furthermore, this study revealed multi-antibiotic-resistant pathogenic bacteria have become increasing threats of otitis media over the last decade in Wollo area, northeastern Ethiopia. By comparing the start and end of the study periods (2001-2 versus 2010-11), the overall resistance rate of all bacterial isolates to one and more of the antibiotics was increased nearly twofold. This finding is higher than studies reported from other African countries and elsewhere in the world [45, 46]. This increasing trend of multiantibiotic resistance rate against middle ear bacterial pathogens such as* Proteus* spp. and* Pseudomonas* spp. calls for more careful attention empirical treatment of middle ear infections in the study area.

Interestingly, our study revealed almost all the isolated pathogenic bacteria were considerably susceptible to ciprofloxacin and gentamicin. Particularly, ciprofloxacin was shown to be highly effective for the three leading pathogenic bacteria associated with middle ear infection in this study:* Proteus* spp.,* S. aureus*, and* Pseudomonas* spp. Likewise, with a slight variation several other authors have shown a similar high efficiency of ciprofloxacin against these bacterial species [5, 15, 17, 47–51]. Thus, we propose that ciprofloxacin can be taken as a first-line optional treatment for middle ear infection in Wollo area, northeast Ethiopia, as it is available in oral, injection, and topical ear drops formulation [48, 51]. This suggestion holds true, since the current first-line treatment for both acute and chronic otitis media in Ethiopia is amoxicillin [52], which already showed a high resistance rate (81.9%) for the majority of the bacterial isolates in this study and in other published data from different parts of Ethiopia [14–19, 53]. Moreover, empiric therapy is advocated when the antibiotic resistance level is 10% or less [54]; however, our finding revealed that the resistance level of most alternative antibiotic treatments for middle ear infection was more than 10%. This implies most common antibiotics used in our study area are no longer appropriate for empiric management of otitis media.

As limitations, the result of this study may not completely represent all the antibiotics used in the clinical practice in the study area since only those who visited the health research laboratory were included in the study and a retrospective study incomplete record and illegible handwriting eliminated some culture results and antibiotics from the study.

## 5. Conclusion

This study revealed that age and the risk of acquiring middle ear infection are strongly associated and* Proteus* spp.,* S. aureus*, and* Pseudomonas* spp. were the three predominant bacteria isolates from patient ear discharges suspected of otitis media. Almost all the isolated bacteria showed a considerable level of resistance to more than one antibiotic that are commonly used in primary health care centers; particularly, majority of isolated bacteria were found to be highly resistant to ampicillin, ceftriaxone, amoxicillin, and tetracycline treatments. More than two-thirds of* Proteus* spp. and* Pseudomonas* spp. have developed resistance to one and more antibiotics used to treat them. A comparative analysis of the overall antibiotic-resistance rate between the start and end of the study periods revealed that the rate of bacterial isolates resistant to one and more of the antibiotics was increased nearly twofold over the last decade in Wollo area, northeastern Ethiopia. This study also indicated ciprofloxacin and gentamicin are effective against all the bacterial isolates and most were highly sensitive to ciprofloxacin. In general, the result of this study revealed that antibiotic-resistant bacteria are alarmingly increasing in Wollo area, the northeastern part of Ethiopia, and becoming a major public health problem in the management patients with middle ear infection. Therefore, we strongly recommend nationwide antimicrobial surveillance to make the right recommendation of alternative antibiotics along with strict adherence to antibiotic policy to reduce the spread of drug resistant microbes in the country.

## Figures and Tables

**Table 1:** Age and sex distribution of bacteria positive middle ear discharges diagnosed from 2001 to 2011 in Dessie Regional Laboratory (*n* = 1024).

Variables	Frequency of bacteria positive ear discharges Number (%)	*χ* ^2^	*P* value
Sex			
Men	516 (50.4)		
Women	503 (49.1)		
Unspecified	5 (0.5)		
Age group in years		45	*P* < 0.001
<5	153 (14.9)		
5–15	253 (24.7)		
16–35	434 (42.4)		
36–50	105 (10.3)		
≥51	47 (4.6)		
Unspecified	32 (3.1)		

*χ* ^2^: chi-square test; *P* < 0.001 implies in each year of the study period “age” has a significant influence on developing bacterial middle ear infection.

**Table 2:** Ten-year retrospective analysis of bacterial isolates from ear discharges.

Bacteria isolated	Frequency	Percentage (%)
*Proteus *spp.	324	28.8
*S. aureus*	266	23.7
*Pseudomonas *spp.	193	17.2
*E. coli*	184	16.4
*Citrobacter *spp.	66	5.9
*Enterobacter *spp.	53	4.7
*Klebsiella *spp.	32	2.8
*S. epidermidis*	5	0.4
*S. pneumoniae*	1	0.1

**Total**	**1124**	**100**

**Table 3:** Overall antibiotic susceptibility profiles of isolated bacteria from ear discharge.

Type of antibiotics used	Frequency of each antibiotic tested	Susceptibility patterns	
		Resistant	Sensitive
		Number (%)	Number (%)
Amoxicillin	491	402 (81.9)	89 (18.1)
Tetracycline	860	641 (74.5)	219 (25.5)
TMP-SMX	684	367 (53.6)	317 (46.4)
Cephalothin	213	96 (45.1)	117 (54.9)
Ampicillin	106	94 (88.5)	12 (11.5)
Chloramphenicol	698	353 (50.6)	345 (49.4)
Ceftriaxone	1319	1115 (84.5)	204 (15.5)
Ciprofloxacin	501	30 (6)	471 (94)
Doxycycline	206	147 (71.4)	59 (28.6)
Erythromycin	634	438 (69.1)	196 (30.9)
Gentamicin	984	169 (17.2)	815 (82.8)
Cefotaxime	396	245 (61.9)	151 (38.1)
Kanamycin	129	50 (38.7)	79 (61.3)
Nitrofurantoin	107	23 (21.5)	84 (78.5)
Vancomycin	120	72 (60)	48 (40)
Carbenicillin	49	22 (44.8)	27 (55.2)
Clindamycin	124	36 (29)	88 (71)

Total	7621	4299 (56.4)	3322 (43.5)

**Table 4:** Antibiotic susceptibility profile of pathogenic bacteria isolated from ear discharges at Dessie Regional Health Research Laboratory from 2001 to 2011.

Antibiotics	Bacterial isolates																				
	*Proteus* spp.			*S. aureus*			*Pseudomonas* spp.			*E. coli*			*Citrobacter* spp.			*Enterobacter* spp.			*Klebsiella* spp.		
	*T*_*t*_	*S*%	*R*%	*T*_*t*_	*S*%	*R*%	*T*_*t*_	*S*%	*R*%	*T*_*t*_	*S*%	*R*%	*T*_*t*_	*S*%	*R*%	*T*_*t*_	*S*%	*R*%	*T*_*t*_	*S*%	*R*%
Amoxicillin	181	21.5	78.5	94	22.3	77.7	87	8.1	91.9	63	22.2	77.8	24	16.7	83.3	26	7.7	92.3	16	12.5	87.5
Tetracycline	237	7.7	92.3	175	41.7	58.3	164	23.2	76.8	158	36.1	63.9	51	25.5	74.5	51	27.5	72.5	24	25	75
TMP-SMX	221	39.8	60.2	156	70.5	29.5	100	8.0	92	93	73.1	26.9	38	39.5	60.5	40	45.7	54.3	36	27.8	72.2
Cephalothin	54	55.5	44.5	48	83.3	16.7	58	34.5	65.5	26	61.5	38.5	11	4/11	7/11	12	5/12	7/12	4	2/4	2/4
Ampicillin	37	0.5	99.5	16	18.6	81.4	14	4/14	10/14	28	10.7	89.3	3	1/3	2/3	3	1/3	2/3	5	0	5/5
Chloramphenicol	206	25.7	74.3	151	72.2	27.8	148	39.7	60.3	103	62.1	37.9	42	59.5	40.5	40	75.0	25	8	5/8	3/8
Ceftriaxone	967	5.0	95	77	63.6	36.4	110	22.7	77.3	102	54.9	45.1	21	57.1	42.9	25	40.0	60	17	23.5	76.5
Ciprofloxacin	114	93.0	7	102	99	1	134	90.3	9.7	69	91.3	8.7	35	97.1	2.9	38	97.8	2.2	9	9/9	0
Doxycycline	48	14.6	85.4	63	47.6	52.4	44	18.2	81.8	38	31.6	68.4	4	1/4	3/4	2	0/2	2/2	7	1/7	6/7
Erythromycin	136	8.1	91.9	200	68.5	31.5	156	15.4	84.6	87	17.2	82.8	6	1/6	5/6	39	15.4	84.6	10	2/10	8/10
Gentamicin	284	81.0	19	241	85.9	14.1	204	75.5	24.5	128	87.5	12.5	52	98.1	1.9	46	82.6	17.4	29	79.3	20.7
Cefotaxime	155	23.9	76.1	75	46.7	53.3	53	26.4	73.6	65	64.6	35.4	18	38.9	61.1	13	8/13	5/13	17	47.1	52.9
Kanamycin	51	56.9	43.1	23	78.3	21.7	13	3/13	10/13	30	70	30	6	3/6	3/6	5	4/5	1/5	1	1/1	0
Nitrofurantoin	37	70.2	29.8	40	87.5	12.5	11	11/11	0	14	9/14	5/14	—	—	—	—	—	—	5	3/5	2/5
Vancomycin	16	18.8	81.2	77	58.4	41.6	20	0	100	5	0	5/5	2	0	2/2	—	—	—	—	—	—
Carbenicillin	32	62.6	37.4	2	0	2/2	—	—	—	10	6/10	4/110	2	1/2	1/2	—	—	—	3	0	3/3
Clindamycin	33	78.8	21.2	50	74	26	21	47.6	52.4	16	81.3	18.7	2	1/2	1/2	1	0	1/1	1	1/1	0

*∗* TMP-SMX: trimethoprim-sulfamethoxazole; *T*_*t*_: number of total tests; *S*%: percent of sensitivity; *R*%: percent of resistance; for those samples ≤15, percentage of susceptibility indicated by fractional numbers: *S*% = number of total sensitive/number of total tests (*T*_*s*_/*T*_*t*_) and *R*% = number of total resistance/number of total tests (*T*_*R*_/*T*_*t*_).
